# The effect of a multi-component behavior change technique intervention on medication adherence among individuals on primary prevention statin therapy: a dose-finding protocol

**DOI:** 10.1186/s13063-023-07549-w

**Published:** 2023-08-12

**Authors:** Mark J. Butler, Anne-Marie N. Romain, Rumisha Augustin, Patrick Robles, Ciaran P. Friel, Thevaa Chandereng, Jerry M. Suls, Elizabeth A. Vrany, Frank Vicari, Ying Kuen Cheung, Karina W. Davidson

**Affiliations:** 1 Feinstein Institutes for Medical Research, Institute of Health System Science, Northwell Health, Manhasset, 130 East 59th Street, Suite 14C, New York, NY 10022 USA; 2 Gordon F. Derner School of Psychology, Adelphi University, Garden City, NY USA; 3 Temple University School of Pharmacy, Temple University, Philadelphia, PA USA; 4 Mailman School of Public Health, Columbia University, New York, NY USA; 5 Donald and Barbara Zucker School of Medicine at Hofstra/Northwell, Northwell Health, Hempstead, NY USA

**Keywords:** Adherence, Behavioral change techniques (BCT), Cardiovascular disease (CVD), Cholesterol, Dose finding, Personalized, Personalized trial, Statin

## Abstract

**Background:**

In the USA, the primary cause of death and morbidity continues to be cardiovascular disease (CVD). Numerous trials have shown that statin medication reduces the likelihood of CVD events; it is a cornerstone of CVD prevention. However, studies have also indicated that up to 60% of the estimated 26.8 million Americans prescribed primary prevention statin treatment are nonadherent during the first year. Multi-component behavioral change technique (BCT) therapies have shown moderate promise in improving medication adherence as well as other positive health behaviors (such as physical activity). However, no research has looked at the duration of multi-component BCT intervention needed to result in a clinically significant improvement in statin adherence behaviors. This study aims to determine the necessary dose of a multi-component BCT intervention (defined as duration in weeks) to promote adherence to statin medication among those on primary prevention statin treatment by utilizing the modified time-to-event continuous reassessment method (TiTE-CRM).

**Methods and design:**

The study will utilize the modified TiTE-CRM in 42 participants, recruited in 14 cohorts of 3 participants each. The goal of this analysis is to identify the minimum effective dose (MED) of a multi-behavior change technique (BCT) intervention required to increase adherence to statins by 20% between baseline and follow-up periods. Using the TiTE-CRM method, the dose of the behavior intervention in weeks will be assigned to each cohort based on the performance of the prior cohort. At the end of the study, the intervention dose that has been found to be associated with a 20% increase in statin adherence among 80% of participants assigned to that dose will be identified as the MED.

**Discussion:**

If successful, the current trial will provide additional guidance to researchers and clinicians seeking to increase statin medication adherence using a BCT intervention by identifying the dose (i.e., the duration) of an intervention required to meaningfully increase adherence.

**Trial registration:**

ClinicalTrials.gov NCT05273736. Registered on March 10, 2022. https://www.clinicaltrials.gov/ct2/show/NCT05273736

**Supplementary Information:**

The online version contains supplementary material available at 10.1186/s13063-023-07549-w.

## Background

Cardiovascular disease (CVD) continues to be among the leading causes of death and morbidity in the USA despite the substantial amount of research and intervention conducted to date [1, 2]. Though many factors can help reduce the incidence and severity of CVD, statin therapy has been identified as a mainstay of CVD prevention [3]. Multiple large, randomized controlled trials (RCTs) have demonstrated that statin therapy decreases the probability of CVD events [3–5]. However, many patients with CVD struggle to achieve optimal medication adherence [6, 7]. Of the estimated 26.8 million Americans prescribed primary prevention statin therapy, studies have shown that up to 60% are non-adherent within the first year [8–10]. Nonadherence to chronic CVD therapy is well documented and contributes to increased CVD risk, hospitalization, and mortality [7, 11]. Increasing medication adherence, including adherence to statin medications, is a priority for healthcare systems as it promotes favorable health outcomes for patients living with CVD while lowering the cost burden of the disease on healthcare systems [7, 12].

Behavior change interventions, specifically multi-component behavior change technique (BCT) interventions, have demonstrated modest efficacy for increasing medication adherence [13–15] and other health behaviors, such as physical activity [16–19]. BCTs are defined as the observable, replicable components of behavior change interventions. For example, the BCT of “Goal Setting” is defined as to “set or agree on a goal defined in terms of the behavior to be achieved” [20]. Medication adherence is a complex behavior based on both situational [21, 22], demographic [21, 22], and psychosocial factors [21, 23, 24]. Multi-component BCT interventions benefit from this by using multiple individual BCTs to address different elements of health behavior. In fact, most digital medication adherence interventions are comprised of 2 or more BCTs [13]. However, research seldom examines the dose of behavioral interventions (i.e., the duration or amount of intervention delivered) [25, 26]. Further, no research has examined the dose of a multi-BCT intervention required to produce a clinically meaningful increase in medication adherence.

In this study, we will identify the minimum effective dose (MED) of a multi-component BCT required to increase statin medication adherence among participants on primary prevention statin therapy who are at elevated risk for CVD. The smallest dosage of a certain treatment or intervention necessary to produce a clinically detectable response is known as the MED [27]. The MED will be used in this study to refer to the shortest BCT dose duration that increases the proportion of days adherent to statin medications by 20%; this has been found to be a clinically meaningful increase in statin adherence associated with reduced odds of mortality in prior studies [28, 29]. The intervention will be comprised of 5 BCTs that have been shown to improve health behaviors in general and medication adherence in particular, namely Goal Setting, Action Planning, Self-Monitoring, Feedback, and Prompts/Cues [13–17, 30]. To identify the MED, this study will use a modified form of the time-to-event continual reassessment method (TiTE-CRM). The TiTE-CRM model can be used to distinguish an MED, but it has typically been employed to establish a maximum tolerated dose for pharmacologic drugs. However, recent research has suggested that this method can be beneficial if used to calculate dose response for behavioral interventions [26, 31]. If successful, the TiTE-CRM method will allow us to identify the dose–response to this behavioral intervention while allowing for potential cost reduction, decreased participant burden, and reduced burnout in future interventions seeking to apply a multi-BCT intervention to increase statin adherence.

While the primary goal of this study is to identify the multi-BCT intervention dose required to increase statin adherence, understanding how the intervention works by identifying potential mechanisms of action (MoAs) for the intervention is also important. Despite the importance in understanding why interventions to increase medication adherence work, few studies of behavioral interventions to promote adherence actually measure MoAs [32]. However, behavioral researchers have also identified potential mechanisms by which BCT interventions can affect health behaviors [33–35]. The current trial examines five of these potential MoAs, namely (1) beliefs about capabilities/self-efficacy, (2) behavioral regulation/intrinsic regulation, (3) feedback processes/discrepancy in behavior, (4) motivation, and (5) environmental context and resources/barriers to adherence. Each of these MoAs have either been previously associated with medication adherence or with the BCTs utilized in the current trial [32–34, 36–39].

In addition to the documented benefits of medication adherence on CVD, physical activity level has been linked to bettering the health outcomes for those with CVD [40]. These include beneficial effects of physical activity on cholesterol, blood pressure, blood glucose levels, and weight [41]. While both physical activity and medication adherence demonstrate great benefits in preventing CVD conditions, research has demonstrated that combining statin medication with physical activity can reduce CVD mortality risk more than statin or physical activity alone [27, 29, 42, 43]. Research has shown that interventions focusing on increasing one positive health behavior may lead to increased levels of engagement in other positive health behaviors [44]. Therefore, we will also examine the benefits of this multi-BCT intervention on physical activity.

The primary aim of this study is to determine the MED of a multi-component BCT intervention in increasing adherence to statins by 20%. This study will also examine potential mechanisms for the intervention and the effects of the intervention on physical activity levels as secondary aims. By identifying the MED (i.e., duration) of a behavioral intervention required to produce a clinically significant change in behavior, we intend to utilize results from this study to guide future research and clinical behavioral interventions designed to increase medication adherence among individuals at risk for CVD. Furthermore, the findings of this study may provide healthcare providers with the framework to implement BCT interventions to improve statin adherence and the overall quality of health for those with CVD.

## Methods

### Study design

This is a dose-finding study examining the dose of a multi-BCT intervention required to increase statin medication adherence among individuals on primary prevention statin therapy in the Northwell Health system. Medication adherence will be measured daily over 5 to 14 weeks using a smart pill bottle. To determine the MED to increase adherence to statins by 20% during the baseline and follow-up periods, this dose-finding study will enroll 42 people (in 14 cohorts of 3 participants each). Participants complete a 2-week baseline period, followed by a variable-duration intervention period and a 2-week follow-up period. During the intervention period, participants will receive a multi-component BCT intervention. Intervention duration will vary for each of the 14 cohorts, ranging from 1 to 10 weeks and determined by the TiTE-CRM approach. Upon the completion of the intervention period, participants will engage in a 2-week follow-up period during which they will participate in data collection without any intervention. The primary outcome, change in statin adherence, will be measured by comparing the proportion of days adherent between the baseline and follow-up periods.

Participants will be provided with a Nomi by SMRxT® electronic pill bottle filled with their clinically prescribed statin medication from an affiliated pharmacy. The Nomi smart pill bottle is a component of a medication event monitoring system that tracks medication usage without active participant input and without the use of any downloadable app. The device is a smart bottle that sends information about participant adherence to the SMRxT® cloud over a cellular connection. Information such as weight changes and bottle movement are used to identify when participants take their medication. Participants who previously utilized other methods of remembering their medication (e.g., a pill box labelled with days of the week) will be encouraged to use the Nomi smart pill bottle. Participants will also receive a commercially available, non-NFC Fitbit device and a Fitbit study account with a special identifier generated by the research team with no personal data relevant to the participant. The Fitbit device will record information such as daily steps taken, floors climbed, activity level, amount of sleep, battery life, and projected sleep stage minutes.

The Nomi smart pill container and Fitbit device will continually collect data on medication adherence and physical activity, respectively, throughout baseline, intervention, and follow-up periods.

**Fig. 1:**
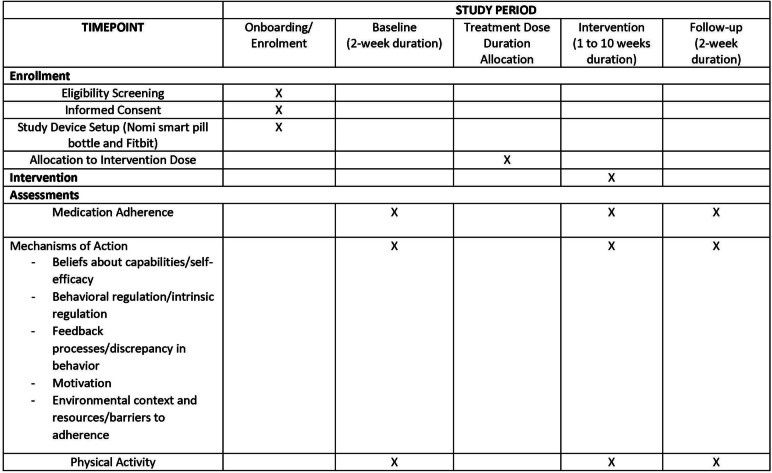
Schedule of Enrolment, Interventions, and Assessments

This trial complies with Standard Protocol Items: Recommendations for Interventional Trials (SPIRIT) reporting guidelines [45]. The schedule of enrolment, interventions, and assessments can be found in Fig. 1.

### Study population

Participants in the study will include adults (age 18 or older) prescribed prevention statin therapy who exhibit low levels of adherence to statin medications. Full inclusion and exclusion criteria are listed in Table 1. Up to 100 participants may be recruited in this study with the intention of assigning 42 participants to the study intervention (14 cohorts of 3 participants each).

**Table 1:** Inclusion and exclusion criteria

Inclusion criteria	Exclusion criteria
Participants must meet the following criteria to be included in the study: • Is aged 18 or older • Is ambulatory without limitations; has never been advised by a clinician that increasing low-intensity walking would be unsafe • Is prescribed statin medication • Has self-reported low levels of adherence to statin medications • Has access to and is capable of using a smart cellular phone • After 2-week baseline, exhibits objectively verified low levels of adherence to statin medications (< 80% of days using statin as prescribed) by Nomi electronic pill bottles	Persons who meet the following criteria will be excluded: • Is aged less than 18 years • Is non-ambulatory or unsafe/not recommended to participate in a walking program • Is not prescribed statin medication • Has a history of CVD • Is unable to comply with study protocol during 2-week baseline • Does not speak English • Is unavailable for follow-up • Has cognitive impairment • Has severe mental illness (e.g., bipolar disorder or schizophrenia) • Is pregnant

### Inclusion criteria

#### Recruitment

The main recruitment methods for potential participants will be advertising and posting on popular Northwell employee networks and sending direct emails to potentially eligible patients taking statin medication identified using the Northwell Health electronic health record (EHR). Potential participants will also be recruited through digital and print media, such as postings in newspapers, social media sites, and websites. Interested individuals will be directed through recruitment links to an online screening questionnaire that covers the inclusion and exclusion criteria for the study and who to contact for more information.

### Consent

Eligible participants will be directed to a short video describing the study protocol and will be given an electronic copy of the consent form to review. The informed consent form will contain a written explanation of the purposes, procedures, and risks of this study in a language appropriate for the individual’s level of understanding. A 4-question assessment measure will assess participant understanding of the protocol and consent process. Once completed, consent will be obtained electronically via web-based software, REDCap, and a copy of the consent will be emailed to the participant for their personal records. Participants may contact a member of the research team with questions about the research at any point in time. Both the research phone and email inbox will be monitored daily by consenting coordinators.

Signed consent forms will be stored electronically on a Health Insurance Portability and Accountability Act (HIPAA)-compliant, Northwell Health-approved shared drive accessible only to the institutional review board (IRB)-approved study staff. An example consent form can be found in Additional file 1. Prior to receiving study devices, participants will read and sign a device allocation document letting them know which devices they may keep upon study completion and which devices should be returned to study staff.

### Onboarding and baseline assessment

After a participant has consented, they will be enrolled into the study and will complete baseline questionnaires. Participants who are employed in the Northwell Health system will receive their medications from VIVO Health, a pharmacy partner of the Northwell Health that can distribute prescriptions via mail. There will be ongoing coordination between the research staff at Northwell, the pharmacy staff at VIVO, and the team at SMRxT®, who will coordinate distribution of the Nomi smart pill bottle. After enrollment but before baseline procedures begin, participants’ information will be provided to VIVO Health, and a participant record will be created in the Nomi online portal by study team members. At VIVO, a pharmacist will fill the participant’s prescription using a Nomi pill bottle sent to them by Nomi in advance. Before sending the pill bottle to the patient, VIVO pharmacists will use the Nomi online portal to link the participant’s study ID with the pill bottle containing the participant’s medication. With the Nomi portal linked to the device, the bottle is shipped to the patient according to the address on file with the pharmacy.

Participants who are not Northwell employees will receive their smart pill bottle in the mail from the study team. Prior to mailing the Nomi smart pill bottle, study staff will use the Nomi online portal to link the pill bottle to the participants’ study ID. Once participants receive their Nomi smart pill bottle in the mail, they will text the Nomi portal and will be provided step-by-step text message guidance to fill the Nomi smart pill bottle with their existing statin prescription. Study staff will be available to answer participant questions and assist them with filling the bottle. All participants will also be mailed a commercially available Fitbit device and will be asked to download the Fitbit app to their phone with the provided username and password.

Participants will begin the trial with a 2-week baseline period during which their pre-intervention levels of adherence to statin medication (assessed using the Nomi smart pill bottle) and compliance with study protocol will be assessed. Participants will also complete one questionnaire at the end of their baseline period to assess MoAs and will have their physical activity levels measured using a Fitbit device. Following completion of the 2-week baseline period, medication adherence of participants will be assessed. If participants are adherent to taking their statin medication at least 80% of the time, they will be withdrawn from the study. Those with adherence that falls below the 80% threshold will receive a BCT intervention, with the length of their intervention period determined by utilization of the modified TiTE-CRM design (i.e., 1–10 weeks).

### Assignment of interventions/treatment randomization

Following the 2-week baseline period, participants will receive a BCT intervention with a variable duration between 1 to 10 weeks, the length of which to be determined using TiTE-CRM to adjust the dose (i.e., intervention duration) for each cohort given the interpretation of the results from the preceding cohort [46, 47]. Neither participants nor the research team are blinded to dose duration.

**Fig. 2:**
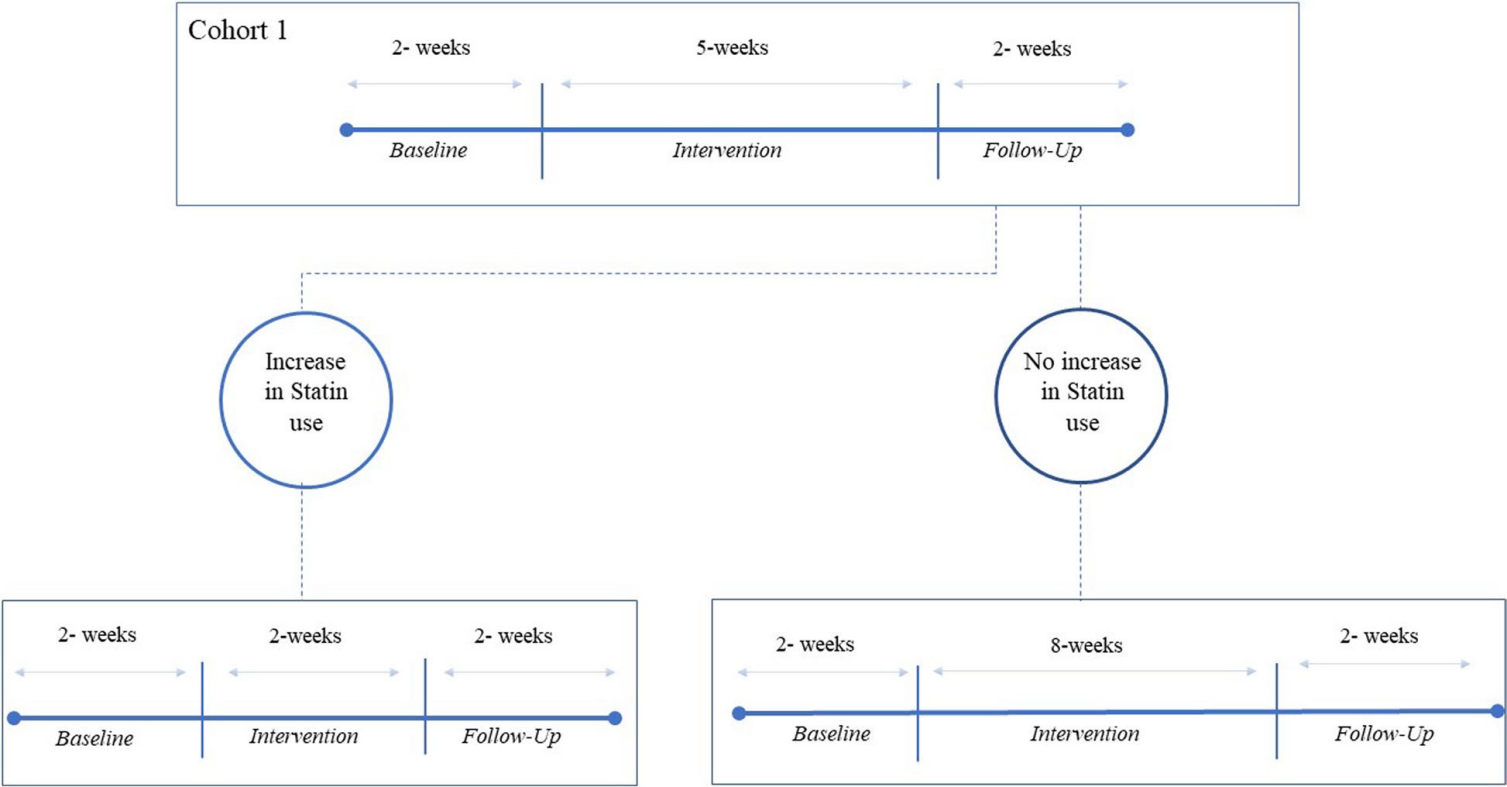
TiTE-CRM dose selection scenario

**Fig. 3:**
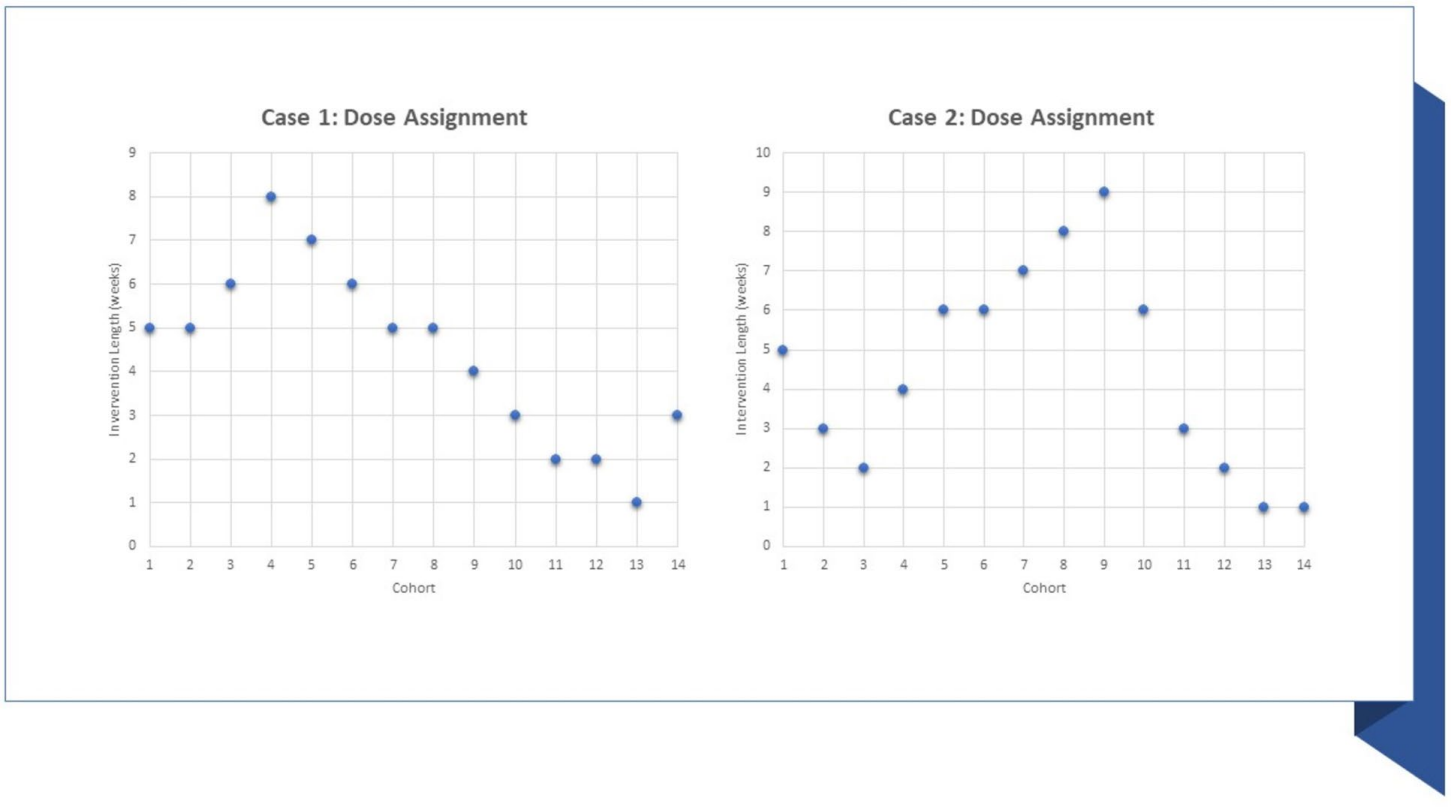
Potential trial dose assignment scenarios using TiTE-CRM. Note: These two cases are hypothetical examples of how doses may be assigned over the course of the trial based on TiTE-CRM methodology. Dose duration can be increased or reduced based on the performance of previous cohorts. Case 1: In this example, participants in initial cohorts have a low frequency of successfully increasing statin adherence by 20%, causing the dose duration to increase initially. However, later cohorts have a greater proportion of successful participants, leading the dose duration to reduce from cohort 9 to cohort 14. Case 2: This example shows an initially high frequency of successful increase in adherence in cohorts 1 through 3. In the next cohorts (4 through 8), the frequency of successful adherence increases is lowered, causing the assigned duration to increase. Once the proportion of participants who increase adherence increases, later cohorts (10 through 14) are assigned lower doses

For the first cohort of 3 participants, the multi-BCT intervention will be administered for 5 weeks. The dose of the intervention given to successive cohorts will vary depending on the initial cohort’s response. An example of the assignment of doses can be seen in Fig. 2. The proportion of participants in the prior cohort who demonstrated the goal behavior (in this case, a 20% increase in statin medicine use between baseline and follow-up periods) will be used by a study statistician to establish the BCT dose for each subsequent cohort. Calculations of dose response to the BCT intervention will depend on comparisons between baseline and follow-up; therefore, participants will not be assigned to successive cohorts until all of the participants in the prior cohort have finished at least 1-week of the intervention period. These interim analyses identifying the dose–response in the trial will be conducted after the completion of each cohort. To further illustrate how the dose–response will work, Fig. 3 shows two hypothetical cases for how doses may be assigned across the 14 cohorts. This figure illustrates how the number of participants who successfully increase their adherence to statin medications may guide the assignment of doses for subsequent cohorts.

### Interventions

Once a participant successfully completes baseline data collection and is found to meet all eligibility criteria, that participant will begin the BCT intervention. The intervention consists of the daily delivery via text message of 5 BCTs that have previously been shown to be effective for increasing positive health behaviors: Goal Setting, Action Planning, Self-Monitoring, Feedback, and Prompts/Cues [13, 16, 17]. Goal Setting involves setting a goal defined in terms of the behavior to be achieved. In this case, participants will receive a text message that asks, “Do you plan to take your medication as prescribed tomorrow? (Please reply YES or NO)”. Action Planning includes detailing the plan of where, for how long, and at what time medication-taking behavior is going to be performed. Implementing this BCT will encourage participants to act or set a behavioral resolution by forming detailed plans that link the behavior to specific situational cues. For example, participants will receive a text that reads, “Think about when and where you will take your medication tomorrow (Have you done this?)”. Self- monitoring of behavior is defined as monitoring and recording behavior. In this aspect, a participant may be asked “Did you remember to take your medication? (Please reply YES or NO)”. Feedback on behavior is defined as providing informative or evaluative feedback on the performance of the behavior. In order to implement this, participants will receive feedback on a previously set goal. The BCT message will be modified based on whether or not participants fulfill their intended goal of taking their statin medication. The text messages will say, “It appears you took/did not take your medication as prescribed yesterday. Is this correct? (Please reply YES or NO)”. Prompts/cues are defined as introducing or defining environmental and social stimuli with the purpose of prompting or cueing behavior. For instance, participants will be sent a text reminding them to take their medication: “Please remember to take your medication soon.”

### Participant timeline

**Fig. 4:**
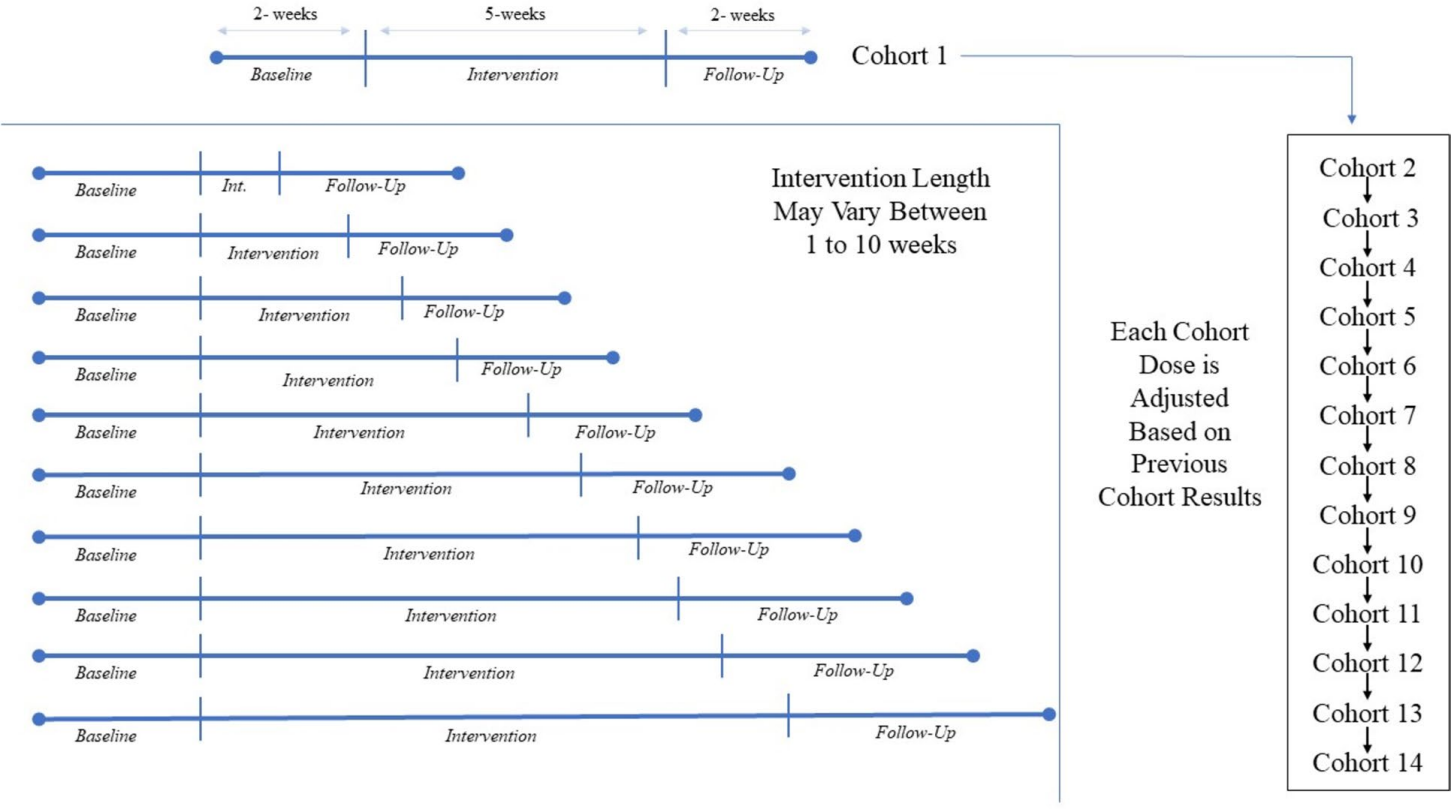
Participant timeline

All participants will undergo a 2-week baseline period and will then be enrolled in cohorts of 3. The first cohort will be given BCT intervention with a duration of 5 weeks. Subsequent cohorts will be given a BCT intervention duration (from 1 week to 10 weeks) based on the updated MED estimate according to the modified TiTE-CRM model and data from the previously enrolled participants. The process will continue until we reach a total of 42 participants (14 cohorts) randomized to the intervention; see sample size justification below. Figure 4 illustrates the participant timeline.

## Outcomes

### Primary outcome

In this study, the primary aim is to identify the required dose of a multi-BCT intervention to increase adherence to statin medication among participants on primary prevention statin therapy with elevated risk for CVD. More specifically, the aim is to determine the MED of a multi-BCT intervention needed to successfully increase adherence to statins by 20% between baseline and follow-up periods in 80% of participants who are assigned a particular dose level (e.g., a 6-week duration). The primary outcome will be determined by comparing the proportion of days of adherence to statin medications during baseline versus the proportion of days of adherence during follow-up, with a 20% increase between baseline and follow-up periods being used to define success.

Participant medication adherence will be monitored using the Nomi™ by SMRxT® real-time medication adherence system. The Nomi™ system includes a smart bottle that tracks and records event information such as weight changes and bottle movement via cellular connection. Adherence to statins will be defined using changes in weight of medication in the Nomi™ smart pill bottle. Average adherence levels in the follow-up period will be compared to average adherence levels in the baseline period. If the proportion of days of adherence to statin medications in the follow-up increases by 20% when compared to the baseline period, the outcome for the TiTE-CRM will be judged successful. The MED will be defined as the smallest BCT dose duration associated with 80% of participants receiving that dose having a successful increase in statin adherence between the baseline and follow-up periods

### Secondary outcomes

Secondary outcomes for the current study include within-participant changes in statin adherence, changes in potential MoAs for the BCT intervention, and any additional benefits of the BCT intervention on physical activity. The primary outcome involves examining success or failure of the intervention (defined by a 20% increase in daily adherence between baseline and follow-up). We are also examining within-person change in adherence as a secondary outcome.

This study will assess 5 potential MoAs by which BCTs may influence adherence to statin medications. The specific MoAs examined in the current study are (1) beliefs about capabilities/self-efficacy, (2) behavioral regulation/intrinsic regulation, (3) feedback processes/discrepancy in behavior, (4) motivation, and (5) environmental context and resources/barriers to adherence. Each of these 5 potential MoAs will be assessed at the end of baseline and every 2 weeks thereafter until the completion of the trial.

Self-efficacy is defined as beliefs about one’s ability to successfully perform a behavior. Self-efficacy will be measured using an adapted version of the PROMIS Item Bank v1.0, Self-Efficacy for Managing Chronic Conditions, Managing Medications, and Treatment Short Form 8a [48]. In this study, we will use a 7-item version measure assessing patient’s capabilities to take their medications and will remove one item relating to management of chronic conditions. Items are scored on a scale of 1 (“I am not at all confident”) to 5 (“I am very confident”) and summed to create a total score. Behavioral regulation is defined as behavioral, cognitive, and/or emotional skills for managing or changing behavior. Behavioral regulation will be assessed using an adapted version of the 4-item Self-Report Behavioral Automaticity Index (SRBAI) [49], which assesses automaticity of behavior. This includes assessing the environment and translating desires into plans. Items are scored on a 1 (“Strongly Disagree”) to 7 (“Strongly agree”) scale and summed to create a total score, with higher scores indicating greater behavioral regulation capability. Feedback processes is defined as processes through which current behavior is compared against a particular standard. Feedback processes will be assessed with a single item adapted from Curtin and colleagues that asks, “How large is the difference between your current medication-taking behavior and the frequency of medication use prescribed by your doctor?” [50]. This item is rated on a scale of 1 (“Not at all different”) to 7 (“Very different”), with higher scores indicating greater levels of discrepancy in behavior. Motivation is defined as processes relating to the impetus that gives purpose or direction to behavior, and it operates at a conscious or unconscious level. Motivation will be assessed with messaging stating, “I feel motivated to take my statin medications exactly as my doctor prescribed.” Motivation will be rated on a scale of 1 (“Not at all true”) to 7 (“Very True”), with a higher score indicating greater levels of motivation. Barriers to adherence is defined as aspects of a person’s situation or environment that discourage or encourage the behavior. This MoA will be assessed using a list of 4 potential environmental barriers to medication adherence taken from Fung and colleagues [51]. Barriers are rated on a 1 (“Not often at all”) to 5 (“Very often”) scale and summed to create a total score, with higher scores indicating that the listed barriers had greater effects on patient nonadherence to statins.

Participant physical activity, defined by daily step count, will be measured with a Fitbit device. In order to investigate any potential correlation between physical activity and adherence, the Fitbit device will be used to determine whether the BCT intervention increases a participant’s levels of activity.

An additional study aim will be to identify participant heterogeneity in the amount of time required to reach a successful increase in statin adherence (defined as an increased proportion of days adherent to the medication over a 2-week period compared to baseline). Average adherence levels will be calculated for each 2-week block during the intervention and follow-up periods. Average adherence levels in these blocks will be compared with the average adherence levels in the baseline period. Once a successful increase has been detected, the time to achieve this treatment response will be recorded. Differences in duration to successful increases in statin adherence will be examined between participants using mixed effects regression models.

## Analysis

### Sample size calculation

The sample size of 42 participants (randomized to complete all study procedures) was chosen to have a sufficient number of participants to obtain a preliminary assessment of the MED for the 5-component behavioral change intervention to increase statin adherence between baseline and follow-up periods. Data will be reported transparently so that individual-level heterogeneity can be assessed.

The dose-efficacy model is calibrated such that the modified TiTE-CRM will eventually select a BCT duration *associated* with 75% to 85% successful statin adherence success, i.e., within 5 percentage points of our target [52]. The sample size (*n* = 42) is determined to achieve 60% probability of correct selection (PCS) under logistic dose–efficacy curves with slope = 0.69 (i.e., an odds ratio [OR] = 2) [53]. As this is small-scale study, power and sample size will be calculated solely for the primary aim.

### Primary analysis

The MED will be defined as the smallest BCT dose duration associated with successful statin adherence increase between the baseline and the follow-up periods among 80% of participants assigned to that dose. For the current study, a successful adherence increase is defined as average daily adherence to statin medications in the 2-week follow-up being higher by 20% or more than in the 2-week baseline. Once the study has completed enrollment, we will identify which dose of the BCT intervention (in weeks) is associated with ≥ 80% of participants achieving the goal of an average of a 20% increase in statin medication adherence between the baseline and follow-up periods. For participants who are non-adherent to the trial or withdraw early, all available data will be utilized to calculate the MED. For example, if a participant is assigned to receive an 8-week intervention but leaves the trial after 4-weeks, all available data will be used to examine the participant’s response to the intervention.

### Secondary analyses

Means and standard deviations of statin use using Nomi™ pill bottles for baseline versus behavioral change strategy treatment period will be visualized using a column graph. The statistical significance of differences will be adjusted for time intervals. The effects of treatment on statin use will be assessed using generalized estimating equations (GEE) with an unstructured variance–covariance matrix for measures on the same day. This model accounts for possible autocorrelation and linear trends between statin use across time.

To identify estimates of the indirect effect of the BCT intervention on adherence via potential mediator MoAs, we will conduct the analysis in 3 steps using mixed effects regression models. First, we will estimate the direct effect of the BCT intervention on adherence. Second, we will estimate the effect of the BCT intervention on each potential MoA. Thirdly, we will estimate the mediation effect of the BCT intervention on adherence via potential MoAs using natural effects models for effect decomposition into direct effect and indirect effect, mediated by increase in each MoA, *relative* to baseline.

The effect of the multi-BCT intervention on physical activity will be examined on the change in participant steps across the study duration. Participant steps will be assessed continuously using a Fitbit wearable device. Daily steps for participants will be aggregated by baseline and follow-up periods to generate average daily steps in each period. Changes in daily steps between run-un and intervention periods will be compared using generalized linear mixed model analyses. Fixed effects will be specified for the intervention, time, and a time-by-treatment interaction, and a random effect will be specified for participants.

Participant variability in amount of time required to reach a successful increase in medication adherence (defined as an increase of 20% in taking statin medication as prescribed over a 2-week period relative to the baseline period) will be examined. The proportion of days taking statin medication as prescribed will be calculated for each 2-week block during the intervention and follow-up periods. The proportion of days of adherence in these blocks will be compared with the proportion of days of adherence in the baseline period. Once a successful increase has been detected, the time to achieve this treatment response will be recorded. Differences in duration to successful increases in statin adherence will be examined between participants using mixed effects regression models.

## Discussion

Despite significant advancements in treatment, with a widespread prevalence and a poor prognosis, the burden of CVD remains high in the USA (Frishmen, 2007). One potential way to ease this burden is by increasing adherence to statin medications that have been proven to reduce cholesterol and help prevent incidence of CVD. Though behavior change interventions have been shown to increase adherence to statin medications [14, 54], researchers are seldom provided with guidance regarding how long an intervention must last in order to encourage a meaningful increase in adherence. This study aims to address this gap by determining the necessary duration of a multi-component behavioral intervention required to increase statin medication adherence with the long-term goal of reducing CVD risk.

However, the current study has two potential limitations. Firstly, the multi-component BCT intervention utilized in the current trial is designed to address particular causes of non-adherence to statin medications. Other common potential causes of nonadherence, such as comorbid health conditions and difficulty accessing medical care [51, 55, 56], are not addressed by the intervention. However, unintentional adherence is significant cause of statin non-adherence [51], suggesting that identifying the dose–response between behavioral interventions and medication is an essential and necessary way to increase adherence to statin medications. Secondly, the current trial utilizes a maximal intervention duration of 10 weeks. It is possible that a longer duration may be needed to influence medication adherence. If we are unable to identify the MED of the BCT intervention utilized in the current trial, we plan to conduct additional trials with longer duration interventions.

These findings will be applicable to many other sectors of research and clinical practice by identifying a specific dose of a well-defined multi-BCT intervention that improves statin adherence. By assessing potential mechanisms of the intervention, the current study will help to identify the manner in which this multi-BCT intervention increases statin adherence and thereby expands knowledge regarding the MoAs. We will also identify additional benefits of the intervention on physical activity and as well as any significant variability in individual participants’ responses to treatment. Overall, the findings will provide doctors and researchers with guidance and tools for improving statin medication adherence and, subsequently, health outcomes in patients at risk for CVD.

## Trial status

The current study protocol is version 3, approved by the Northwell Health Institutional Review Board (IRB) on May 2, 2023. Trial recruitment began on July 21, 2022, and is anticipated to complete recruitment by May 31, 2023. Any changes to the trial protocol will only be implemented following approval by the Northwell Health IRB. Protocol changes will also be reflected in the trial’s informed consent and by updating the trial on clinicaltrials.gov.

## Patient and public involvement statement

Pilot data with participants was used to help determine which interventions were selected for the current trial. We did not directly involve participants in any other elements of the design or conduct of this trial.

## Data monitoring

Since the study activities involve no more than risks encountered in daily life (i.e., taking medication already prescribed by a physician), the study has received approval from the National Institute on Aging (NIA) for a safety monitor. Dr. Zenobia Brown has been approved to provide safety monitoring for this study. Dr. Brown is a family medicine clinician and oversees Northwell’s Health Solutions programs. Also, given that study activities involve no more than risks encountered in daily life (e.g., increased time spent walking by healthy, working individuals), the study has not convened a data and safety monitoring board (DSMB).

## Harms

### Treatment adverse events

This study poses low risk of physical harm to subjects. One risk of taking part in this study is the possibility of a loss of confidentiality or privacy. The study team plans to protect privacy by only sharing necessary information about participants to those outlined in the consent form. All subjects will be informed that their responses are confidential and that they may refuse to participate in the project or withdraw at any time without explanation, and that such action will not affect their future interactions with their health care providers, employment, educational studies, or the research study. The risk of loss of confidentiality will be reduced by minimizing use of protected health information (PHI) and securely storing data that includes PHI in a Northwell-approved database.

Using a Fitbit activity monitor for research as compared to using the device as a consumer presents no additional risk, including mild skin irritation (i.e., contact dermatitis) that occurs among a small proportion of users. Participants will be instructed via the consent form on methods to reduce irritation (i.e., how to keep the band clean and dry) and that they can remove the band for a short period of time.

No known risks are associated with utilization of the BCTs employed in this intervention. Statin use is not research driven, but rather clinically indicated and prescribed prior to enrollment in the research. As such, risks of statin use are not considered research risks. Expected benefits and harms of statin use would have been assessed by the clinical provider prior to prescribing use of statins. We will emphasize to participants that although the aim of the research is to increase their statin adherence, this is not a treatment protocol. The informed consent document will clarify that as the aim is to assess the behavioral intervention that increases adherence, they should expect to experience side effects previously discussed with their prescribing physician.

No known risks are associated with the utilization of the Nomi™ medication adherence vial. The vial cap meets all federal stands for safety (i.e., it is childproof). The Nomi™ vial has no GPS tracking. The proposed questionnaires are not anticipated to pose risk to the participants. Participants will receive text message notifications with a secure link to a survey that can be accessed via a smartphone. All survey responses will be directly entered by participants in an electronic format (i.e., a secure, HIPAA-compliant REDCap database).

### Costs

This research study is funded by the National Institutes for Health (P30AG063786-01). All study-related devices will be provided to participants at no cost. Participant insurance will not be billed. This study uses text messaging to deliver notifications, reminders, and study questionnaires. Standard message and data rates from the participant’s wireless carrier may apply to the study participant. Study participants will not be compensated for any costs related to data usage or sending or receiving text messages by the study or by members of the study team.

## Compensation

Participants will be compensated $110 and will be allowed to keep their commercially available Fitbit Versa 2™ (a value of up to $150.00) for participation in this research. Participants will be required to return their Nomi™ smart pill bottle at the end of the research study. If participants do not return the pill bottle at the end of the study, participant compensation will be reduced by $110 to compensate for the loss of the smart pill bottle but will still be allowed to keep their Fitbit Versa 2™.

## Dissemination

The trial results will be published in a peer-reviewed journal. All publications resulting from this series of personalized trials will follow the CONSORT reporting guidelines. Trial results will be reported to study collaborators and participants following study completion.

### Supplementary Information

**Additional file 1.** Consent for Participation in a Research Study.

## Data Availability

De-identified data for the current study will be published on the Open Science Framework. Data requests for potentially identifiable data will be reviewed by the regulatory team and access to full data will be granted following Institutional Review Board (IRB) approval, as applicable, and completion of a data use and sharing agreement with Northwell Health.

## Abbreviations

BCT: Behavioral change technique
CVD: Cardiovascular disease
DSMB: Data and safety monitoring board
HER: Electronic health record
HIPAA: Health Insurance Portability and Accountability Act
MED: Minimum effective dose
NIA: National Institute on Aging
PHI: Protected health information
RCT: Randomized controlled trial
SRBAI: Self-Report Behavioral Automaticity Index
TiTE-CRM: Time-to-event continual reassessment method
MoA: Mechanism of action
