# Supplementary material for: The effect of a multi-component behavior change technique intervention on medication adherence among individuals on primary prevention statin therapy: a dose-finding protocol
Source: Trials. 2023 Aug 12;24:523. doi: 10.1186/s13063-023-07549-w (PMC10422706; doi:10.1186/s13063-023-07549-w)
Supplement: Supplementary file 1 — Additional file 1. Consent for Participation in a Research Study. [file 13063_2023_7549_MOESM1_ESM.docx]

**Northwell Health**

**Consent for Participation in a Research Study**

**Study Title:** The Effect of a Multi-Component Behavior Change Technique Intervention on Medication Adherence Among Individuals on Primary Prevention Statin Therapy: A Dose-Finding Pilot Study

**Principal Investigator:** Mark Butler, PhD

**IRB Protocol:** 21-0707

**Version Date:** 1/19/2023

**About this research**

You are being asked to participate in a research study. This consent form will give you information about the study to help you decide whether you want to participate. Please read this form, and ask any questions you have, before agreeing to be in the study.

**Important Information**

This information gives you an overview of the research. More information about these topics may be found in the pages that follow.

| **Why am I being asked to provide my consent?** | This is a research study, which is different than personal medical care. Scientists do research to answer important questions which might help change or improve the way we do things in the future. |
| --- | --- |
| **Do I have to join this research study?** | No. Taking part in this research study is voluntary. You may choose not to take part in the study or may choose to leave the study at any time. Deciding not to participate, or deciding to leave the study later, will not result in any penalty or loss of benefits to which you are entitled. |
| \| **Why is this research study being done?** \|  \| \| --- \| --- \| | We would like to find out what duration of a multi-component behavioral intervention will increase your statin medication use (adherence). The long-term goal is to prevent cardiovascular disease (CVD). |
| **What will happen to me during the study?** | Participation involves completion of questionnaires, wearing an activity monitor (e.g. Fitbit) continuously throughout the study, using an electronic pill bottle to track statin use, and participation in a multi-behavior change technique (BCT) intervention for a defined amount of time. More information about study procedures can be found below. |
| **How long will I participate?** | The study may last up to 14 weeks. All participants undergo 2 weeks of baseline procedures before participating in the intervention phase and 2 weeks of observation after participation in the intervention phase. Behavior change technique participation (the intervention) lasts from 1 week – 10 weeks, depending on your group assignment. In other words, the minimum duration is 5 weeks, while maximum duration is 14 weeks. More information about the length of study participation can be found below. |
| **Will taking part expose me to risks?** | This study poses low risk to participants. A potential risk of taking part in this study is the possibility of a loss of confidentiality or privacy. You may also experience skin irritation from the activity monitor band. Some of the questions we ask in the survey are personal. You may feel embarrassed or stressed. Expected harms of statin use were assessed by your clinical provider prior to prescribing the medication and are not considered research risks. |
| **Are there any benefits to participation?** | Participants may receive a direct benefit of increased statin use. The information collected from participant involvement will inform the development of future research to help other participants and eventually patients discover which behavioral treatment options are best for them as an individual. |
| **What are my alternatives to participation?** | The alternative is not to participate. |

**Please review the rest of this document for details about these topics and additional things you should know before making a decision about whether to participate in this research. You should ask questions before you decide if you want to participate. You can also ask questions at any time during the study:** statincohort@northwell.e**du**

**Introduction and Research Purpose**

You are being asked to join a research study. The purpose of a research study is to answer specific questions. In this research, we will study 5 Behavior Change Techniques, or BCTs, which have previously been shown to be effective on increasing healthy behaviors: Goal Setting, Action Planning, Self-Monitoring, Feedback, and Prompts/Cues. We would like to find out which BCT package (length of intervention) will increase your statin medication use (adherence).

You do not have to be in this study to receive medical care if you are a Northwell Health patient, and your decision to participate or not participate will not affect your employment at Northwell Health if you are a Northwell employee.

**Why am I being asked to participate?**

You are being asked to participate in this study because you are a person who is currently at risk for cardiovascular disease, have been prescribed statin medication by your clinical doctor and are interested in increasing your statin medication use (adherence).

**How many people will take part in this study?**

This research study hopes to enroll up to 100 people in this study, with the goal of having at least 42 people randomized to the intervention phase. Randomization means that you are put into a group by chance. It is like flipping a coin.

**How long will I be in this study?**

If you choose to take part in this study, your participation may last up to l4 weeks. See below for more information on how participation duration is calculated and what this means for you.

**What will happen in this research study?**

After filling out this consent form, you will be asked to provide the research team with some more information about yourself. This will include your cell phone number, cell phone carrier, home address, and information about your statin prescription (including name of medication and dose). This pilot study uses text message prompts and reminders to help you through the study protocol. Information about your statin prescription will be used to help fill your medication through Vivo Health pharmacy if you use Vivo Health pharmacy.

This protocol involves the following phases:

- Baseline
- Intervention
- Observation

**Baseline:** You will be mailed study devices to start your baseline period. Prior to being sent these devices, we will ask you to sign a form that acknowledges the devices that you will receive and when you should send them back. When you receive your Fitbit activity monitor, you will be asked to download the Fitbit app to your personal phone in order to use the Fitbit and will be asked to wear the activity monitor continuously for two weeks and continue physical activity as you normally do each day. If you are a Northwell employee who receives their prescriptions from Vivo Health, you will also be mailed an electronic Nomi wireless pill bottle that has been filled with your clinically prescribed stating medication by the Vivo Health Pharmacy. If you are not a Northwell Health employee, or do not use Vivo Health Pharmacy, you will be mailed an empty Nomi pill bottle from the study team. You will then receive instructions that ask you to send a text message to Nomi, and they will provide you instructions on filling the bottle with your own statin medication. Study staff will be available to support you if you have questions about filling the NOMI bottle with your statin medication. No study team member or member of the SMRxT/NOMI team will have direct contact with your prescription medication.

There is no app to download for Nomi, but you will also be asked to agree to receiving text messages from Nomi as part of the intervention. If you do not agree to receiving these text messages, you will not be able to participate in the study. You will be asked to continue to take your medication as you normally do. At the end of your baseline period you will be asked to complete one questionnaire. If you pass baseline, you will keep the Nomi pill bottle and move on to the next phase described below. There may be a 1 - 2 day delay before you are assigned to an intervention group. If you do not pass baseline, you will be asked to return the Fitbit activity monitor and the Nomi pill bottle.

**Intervention**: During the intervention period you will continue to wear your activity monitor, use the Nomi pill bottle. You will also be assigned an intervention BCT package to follow for a defined period of time. During the BCT intervention, all participants will receive 5 daily BCTs via text message (BCTs include: Goal Setting, Action Planning, Self-Monitoring, Feedback, or Prompts/Cues) and will complete one questionnaire every two weeks (if applicable according to your BCT package). The first three participants who are eligible to continue to the intervention phase will be assigned to a BCT package that lasts for five weeks. All other participants will be assigned to a group that receives 5 daily BCTs via text message (as described above) but the length of the intervention will be variable (meaning it will change). For instance, if you are not one of the first three people to participate, you will be assigned to a group that is asked to receive a BCT package for 1 week, 2 weeks, 3 weeks, etc. up to 10 weeks in length.

**Observation**: After the BCT intervention period ends, all participants are then observed for another 2 weeks. During this time, participants will continue to wear their activity monitor and will continue to use their Nomi pill bottle until the end of the 2 weeks. At the end of the 2 weeks, participants will be asked to complete the same questionnaire completed during baseline and the intervention phase, as well as one a satisfaction survey (electronic or by phone/teleconference). **You must return the Nomi smart pill bottle at the end of your participation in the research. The smart pill bottle can be returned in a box provided to you. The box and the envelope will all have prepaid labels on them along with shipping instructions.** No personal information, such as your name or address, will be printed on these materials.

In summary, total participation will be between 5 – 14 weeks depending on your BCT dose assignment. During the baseline, pilot study intervention period and observation period, you may also receive additional text messages to ask you to sync your data if it is not appearing correctly.

**What are the risks of the research study? What could go wrong?**

Since the aim of this research is to see which BCT will successfully increase your statin use (adherence), you should expect to experience side effects of statin medication that were previously discussed with your prescribing physician. These expected harms of statin use were assessed by your clinical provider prior to prescribing the medication and are not considered research risks. In addition, some of the questions we will ask you are personal. You may feel embarrassed or stressed. You may ask to see the questions before deciding whether or not to take part in this study. You may also experience mild skin irritation (rash) from wearing the Fitbit band during this research study. To reduce irritation, keep the band clean and dry. To provide relief for you skin if this mild risk occurs, remove the band for a short period of time. Lastly, another potential risk of taking part in this study is the possibility of a loss of confidentiality or privacy.

**What are the benefits of this research study?**

The possible benefits you may experience from the procedures described in this study include a direct benefit of increased statin medication use (adherence). In addition, the information collected from participant involvement will inform the development of future BCTs to help other research participants and eventually patients discover which behavioral treatment options are best for them as an individual.

**Will I receive my results?**

We may learn things about you from the study activities which could be important to your health or to your treatment; however, we will not share this information with you because these results are considered research results and are not considered clinical information.

**If you do not want to take part in this research study, what are your other choices?**

If you do not join this study, you have other choices to help increase your statin use (adherence). Talk to your doctor about your choices. An alternative is not to participate.

**Are there any costs for being in this research study?**

This research study is funded by the National Institutes for Health (NIH). All study related devices will be provided to participants at no cost. Participant insurance will not be billed.

This study uses text messaging to deliver notifications, reminders, and study questionnaires. Completing the surveys will require cellular data if you are not connected to Wi-Fi.  Standard message and data rates from your wireless carrier may apply. Study participants will not be compensated for any costs related to data usage or sending or receiving text messages by the study or by members of the study team.

**Will you receive any payments for participating in this research study?**

You will be able to keep your commercial Fitbit device after you have been randomized to the intervention phase of the research. Y**ou must return the Nomi pill bottle at the end of your participation in the research.** We do not own the Nomi pill bottles used in this research; therefore, future participants depend on their successful return. It is important to be aware of your responsibility to return the pill bottle after your participation in this research ends. **Your participation ends after completion of the satisfaction survey or early withdrawal from the study after you have been randomized to the intervention phase.** We will send you reminders to return the device. After the device is received, you will be sent a ClinCard valued at $110 which is the cost of the pill bottle. No incentive will be provided if the bottle is not returned or if you have not been randomized to the intervention phase.

**What are your rights as a research participant?**
Your participation in this project is voluntary. Your decision to participate or not participate will have no bearing on your performance evaluation or employability at Northwell Health if you are a Northwell employee. If you receive care at Northwell Health, the quality of your medical care will be the same, whether you join, refuse to join, or decide to leave the study. If you do not join the study, you will not be penalized or lose benefits to which you are entitled. I**f you join the study, you may withdraw at any time without prejudice to your future care and/or employability at Northwell Health.**

**Could you be taken off the study before it is over?**

It is also possible that your participation in this study may end without your consent.  This decision may be made by a researcher, study sponsor or the Institutional Review Board (IRB- the committee that oversees research at this institution).

Reasons for withdrawal may include:

- failure to follow instructions, including maintaining less than 80% adherence to survey responses and Fitbit use;
- significant cell phone carrier issues that prevent you from receiving study text messages;
- your prescription medication or dose changes;
- it is not in your best interest to continue on this study, or
- the study is stopped.

If your prescription medication or dose changes during your participation in this study, please contact the study team so that we may ask additional questions to assess if you can continue to participate. If you withdraw from this study or if you are withdrawn from the study, any data already collected will continue to be used.  However, no new data will be collected.  In addition, you will be sent an electronic survey including questions regarding your experience during the study, or will be contacted by phone or teleconference. Completion of this survey is voluntary and will help us design better research in the future.

**What happens if new information is learned?**

You will be told of any new findings that may change your decision to continue to participate. Your consent to continue to take part in this study may be obtained again.

**What will happen with the information we collect as part of this research study?**

If the results of this study are published, your name will not be used. Your research records will be private to the extent allowed by law. In order to make sure the research is done properly, the Human Research Protection Program (the group of people that oversees research at this institution) may need access to information about your participation in this study.

**What information will be collected and used for this study?**

If you agree to be in this study, we will collect health information that identifies you. We may collect the results of questionnaires and behavior change technique/interventions via REDCap, a Northwell approved electronic platform, and using text message responses. Text messages will alert you to a new message from “Northwell Statin Study” and contain a link to open this secure browser directly on your phone. No identifying information will be shared via text message. The study team will have direct access to the survey and Fitbit data, as applicable, through REDCap and Fitabase.

Data collected by the Nomi electronic pill bottle includes weight of the contents of the smart pill bottle. The smart pill bottle will cease measurement at the end of the study. No personal data is collected or stored on the Nomi device. All stored date, time, and weight stamps on the bottle cap will be wiped from the device upon successful return to the study team. Information from the Nomi device will be sent wirelessly to an online data portal. The Nomi online data portal will also contain information about your medication use (including name of statin medication and dosing) and will contain personal information for delivery of the intervention (including your study ID, name and phone number). This information will be deleted from the Nomi online portal following your completion of the study and returning the Nomi smart pill bottle.

We may also collect Fitbit activity data (steps, activities, intensity, heart rate, floors climbed, breathing rate, heart rate, and heart rate variability etc.), sleep data (total sleep minutes, sleep stage estimates, sleep and wake times), and device data (last sync date and Fitbit battery level). You will be asked to download the Fitbit app to your personal phone in order to use the Fitbit device. Your IP address may be collected by the Fitbit app which could be considered identifiable information. Your Fitbit data will be securely stored in Fitbit electronic platforms as is standard for all Fitbit users. Fitbit’s Terms of Service and Privacy Policy are separate from this research consent form and you will need to agree to Fitbit’s Terms of Service and read Fitbit’s Privacy Policy in order to create a Fitbit account using the username and password provided to you via the Fitbit app before you join the study.  Fitbit’s Terms of Service includes information about your legal rights when using Fitbit’s products that may differ from your rights as a participant in this study.  Fitbit’s Privacy Policy describes how Fitbit collects, uses, shares, and protects your data. You can exercise your right to access your personal information by logging into your Fitbit account and using your account settings. Fitbit may also have access to device identifiers so they may be able to identify that you are a participant in this research. For more information about the information that Fitbit may have access to, refer to Fitbit’s Privacy Policy.

Data from your Fitbit device may be shared with Northwell Health for research purposes through an online portal called Fitabase. The study account given to you to connect your Fitbit will be linked to an identification number in the Fitabase system. No information that could be used to identify you will ever be shared with Fitabase. Only the research team will have access to data that will be able to connect a research participant to their Fitabase ID. Fitabase will stop sharing your data at the end of your study, but as an added step, you will be asked to remove the study account from your device if you would like to keep your Fitbit.

We may also collect your email address and phone number, mailing address and demographic information. We will only collect information that is needed for the research. This information has been described in this consent form. If you sign this consent form, you are giving us permission to collect, use and share your health information. This permission is called authorization. If you do not want to provide authorization, then you cannot participate in this research study.

**Who else will see your information?**

Study records that identify you will be kept private. You will not be identified in study records or publications disclosed outside Northwell Health, except as detailed below.

Investigators will share information collected from this research study with:

- Study sponsor (NIH) and/or its agents,
- other researchers,
- accrediting agencies,
- data safety monitoring officer, clinical staff not involved in the study who may be involved in participant's treatment, billing,
- health insurers or payers.

The following reviewers may access your study and medical records to make sure that this study is being done properly:

- Representatives from Federal and state government oversight agencies, such as the Department of Health and Human Services and the NIH, etc.
- Representatives from Northwell Health’s Human Research Protection Program (a group of people that oversee research at this institution)

We will do our best to protect the privacy of your records but it is possible that once information is shared with people listed on this form, it may be released to others. If this happens, your information may no longer be protected by the federal law. In the future, we may publish results of this study in scientific journals and may present it at scientific meetings. If we do we will not identify you. If the researchers learn about potential serious harm to you or someone else or other public health concerns, it will be shared with the appropriate authorities.

If you agree to let us use and disclose your protected health information, we will collect your health information until the end of the research.

**Will you be able to access your records?**

If your research records are used for decisions related to your clinical care, then you have the right to review this information and request changes. This is limited to information about your treatment, and does not include information related to procedures or tests that are for research purposes only. You may access this information only after the study analysis is complete. You have the right to know who has and who will see your records. To request this information, please call the Human Research Protection Program at 516-465-1910.

**How long will your health information be kept?**

There is no limit on the length of time we will keep your information for this research because it may be analyzed for many years. We will keep it as long as it is useful, unless you decide you no longer want to take part or we close the study. You are allowing access to this information indefinitely.

**Can you change your mind?**

If you change your mind about being in the study, you may withdraw at any time. If you want us to stop collecting your health information, you need to send a letter or email to the researcher at the following address:

Dr. Mark Butler, PhD

Institute of Health System Science

130 E 59th Street, Suite 14C

New York, NY 10022

[statincohort@northwell.edu](mailto:statincohort@northwell.edu)

Your letter or email needs to say that you have changed your mind and do not want the researcher to collect and share your health information. You may also need to leave the research study if we cannot collect any more health information. We may still use the information we have already collected. We need to know what happens to everyone who starts a research study, not just those people who stay in it.

**Will information about this study be available to the public?**

A description of this clinical trial will be available on <http://www.ClinicalTrials.gov>, as required by U.S. Law. This Web site will not include information that can identify you. At most, the Web site will include a summary of the results. You can search this website at any time. In addition, we also plan to share information about the pilot study, including de-identified data only, on the following data sharing website: https://cos.io/. The Open Science Framework is a free, open-source web application built to provide researchers with a free platform for data and materials sharing.

**Certificate of Confidentiality**

To help us protect your privacy, this research is covered by a Certificate of Confidentiality from the US Department of Health and Human Services (DHHS). The Certificate of Confidentiality means that researchers cannot be forced to identify you, even under a court subpoena. The Certificate does not mean the Secretary of DHHS approves or disapproves of the project. It adds special protection for the research information about you. You should know, however, that researchers may provide information to appropriate individuals or agencies if harm to you, harm to others or child abuse becomes a concern. A Certificate of Confidentiality does not prevent you or a member of your family from voluntarily releasing information about yourself or your involvement in this research. However, if an insurer, employer or other person learns about your participation and gets your consent to receive research information, then the researchers will have to provide your information.

**Will my information be used for research in the future?**
Information for this research may be used for future research studies, pooled with other personalized trial or Institute of Health System Science participants, or shared with other researchers for future research. If this happens, information which could identify you will be removed before any information is shared. Since identifying information will be removed, there will not be an additional consent for future research. By consenting to participate in this study you are agreeing to allow your coded data to be used by future researchers without additional consent.

Some information collected during this study that can identify you will be kept on file. This information may be used in the future to contact you for future participation in research studies. This information will be stored on a secure database. It will only be accessible by trained members of the study team. If you change your mind about being contacted in the future, you may follow the procedures outlined above to notify the researcher.

**Does the investigator of this study receive money if you take part?**

The investigators on this study receive money to conduct the study, but do not financially benefit from your participation.  The money they receive is to pay them back for the costs of conducting the research study. Funding for this research study is provided by the National Institutes of Health.

**Who can answer your questions about this study?**

If you have any questions about the study, you may contact a member of the study team at [statincohort@northwell.edu](mailto:statincohort@northwell.edu). If you have questions about side effects or injury caused by research, you should call Dr. Mark Butler at (646) 877-7393. If you need emergency care, dial 911 or go to the nearest Emergency Room. If you have questions about your rights as a research participant, concerns about being in the study, or would like to offer input, you may contact the Office of the Institutional Review Board (the committee that oversees research at this institution) at (516) 465-1910.

A signed copy of this consent form will be given to you.

Please answer the following questions about the consent form:

1. As a participant in this study, I will be asked to follow a behavior change technique package that could last anywhere from one week to ten weeks.
   - True (Once selected- Correct! Way to go!)
   - False (Once selected- Incorrect. Please try again.)
2. As a participant in this study, I will be asked to wear the provided Fitbit device at all times during the study, even to sleep.
   - True (Once selected- Correct! Way to go!)
   - False (Once selected- Incorrect. Please try again.)
3. As a participant in this study, I will be asked to answer a questionnaire about the behavior change technique package during baseline, bi-weekly during the intervention phase and at the end of the observation phase..
   - True (Once selected- Correct! Way to go!)
   - False (Once selected- Incorrect. Please try again.)
4. I must return the Nomi pill bottle to the study team at the end of the study.

- True (*Once selected-*Correct! Way to go!)
- False (*Once selected-* Incorrect. Please try again.)

1. If my prescription or medication dose changes during my participation in this study, I must contact the study team so that they can assess if I can still be in the research.

- True (*Once selected-*Correct! Way to go!)
- False (*Once selected-* Incorrect. Please try again.)

[Signature Page Follows]

**Summation/Signature**

You have read the above description of the research study. You have been told of the risks and benefits involved and all your questions have been answered to your satisfaction. A member of the research team will answer any future questions you may have. You voluntarily agree to join this study and know that you can withdraw from the study at any time without penalty. By signing this form, you have not given up any of your legal rights.

____________________________________________________________

Printed Name of Participant

___________________________________________________ ______________

Signature of Participant Date
